# Comparison of Early versus Traditional Rehabilitation Protocol after Rotator Cuff Repair: An Umbrella-Review

**DOI:** 10.3390/jcm12216743

**Published:** 2023-10-25

**Authors:** Teresa Paolucci, Francesco Agostini, Marco Conti, Sara Cazzolla, Elena Mussomeli, Gabriele Santilli, Federica Poso, Andrea Bernetti, Marco Paoloni, Massimiliano Mangone

**Affiliations:** 1 Department of Oral Medical Science and Biotechnology, G. D’Annunzio University of Chieti-Pescara, 66100 Chieti, Italy; teresa.paolucci@unich.it (T.P.); federica.poso@studenti.unich.it (F.P.); 2 Department of Anatomical and Histological Sciences, Legal Medicine and Orthopedics, Sapienza University, 00185 Rome, Italy; francesco.agostini@uniroma1.it (F.A.); ma.conti@uniroma1.it (M.C.); sara.cazzolla@uniroma1.it (S.C.); elena.mussomeli@uniroma1.it (E.M.); marco.paoloni@uniroma1.it (M.P.); 3 IRCSS San Raffaele Roma, 00163 Rome, Italy; 4 Department of Surgical and Medical Sciences and Translational Medicine, Sapienza University of Rome, 00185 Rome, Italy; gabriele.santilli@uniroma1.it; 5 Department of Biological and Environmental Sciences and Technologies (DiSTeBA), University of Salento, 73100 Lecce, Italy; andrea.bernetti@unisalento.it

**Keywords:** rotator cuff, shoulder, rehabilitation, physiotherapy, physical therapy, exercise, pain

## Abstract

Rehabilitation after rotator cuff repair is crucial for functional recovery and for minimizing the risk of retear. There are two rehabilitation protocols (early and traditional) and the debate about which is the best is open. This umbrella review aimed to compare the effect of these rehabilitation protocols in terms of reduction in pain, functional recovery, and retear risk. We selected systematic reviews and meta-analyses published between 2012 and 2022 dealing with the aim. Nineteen systematic reviews were included. No significant differences were found between early and traditional protocols in terms of pain reduction. Early rehabilitation provided better short-term results regarding Range of Motion improvement, but long-term functional outcomes were similar. Retear risk remains a significant concern for the early protocol. We found major differences between the analyzed protocols. This review suggests that both protocols are useful to recover global shoulder function, but the standard protocol has a greater safety profile for larger tears. On the other hand, the early protocol may be preferable for smaller lesions, allowing a faster recovery and having less impact on medical costs. Further research is needed to identify optimal rehabilitation strategies tailored to the individual patient’s needs and characteristics.

## 1. Introduction

Rotator cuff tear is a common cause of shoulder pain, decreased range of motion, and weakness of the upper limb, limiting people’s daily activities such as brushing hair or putting on clothes, caused by repetitive overhead lifting or shoulder injuries [1]. Mechanisms underlying rotator cuff pathology include acute accidents as well as chronic issues, often influenced by repetitive activities or micro traumas, subacromial pain syndrome, and aging; intrinsic factors such as poor vascularity and alterations in matrix composition are also involved [2,3,4]. We focused our research on the rehabilitation of chronic tears, often related to repetitive overhead work activities, which lead to abnormal alterations in rotator cuff tendons [5]. Rotator cuff tears are mostly found in adults and elderly people [6]: around 15–20% of 60-year-olds present this impairment as well as 26–30% of 70-year-olds, and 36–50% of 80-year-olds [7,8]. An optimal alternative to conservative treatment is the rotator cuff surgical repair [9,10], an approach that has been consolidating in recent years: more than 270,000 rotator cuff repairs are performed annually in the United States and around 9000 in the UK [11,12]. The incidence of these procedure has been rising from 1995 [11]; however, the postoperative protocol has not evolved over the last two decades, so it is essential to identify the best rehabilitation approach [13,14]. Concerning surgical techniques, different procedures can be performed: open, mini-open, and arthroscopic repair. Arthroscopy is increasingly becoming the first choice because of less postoperative pain and minor trauma due to smaller incisions through which the operation can be performed with the help of a video display for visual control [15,16]. Despite positive clinical results, reports of structural failure after surgical repair range from 10% to 48.4% [17,18]. For tears larger than 4 cm, failure occurs even more often, up to 94%, especially within the first 3 months after surgery [19,20]. The objectives of post-surgery rehabilitation are shoulder function recovery, tendon healing, and retear risk reduction. Traditionally, it is possible to identify two rehabilitation protocols: the early protocol and the delayed or traditional protocol. The early rehabilitation protocol consists of passive shoulder range of motion exercises, such as pendulum flexion, external rotation, and manual passive exercises; the patient begins these exercises the first postoperative day with a weekly high frequency. Instead, the traditional protocol is frequently based on sling immobilization and no physiotherapy: the only exception is the pendulum exercise performed for 4–6 weeks postoperatively. Another difference is in the beginning of the strengthening exercises that usually start later in the standard protocol [21,22,23]. A reason to prefer the delayed protocol lies in the tendon healing time, usually estimated from 4 to 16 weeks [24]. Table 1 shows a detailed comparison between the two protocols provided by Cuff and Pupello [21], who divided the recruited patients into two groups, early and delayed protocol, undergoing different rehabilitation programs. 

The evidence suggests that the early protocol may prevent postoperative stiffness, fatty infiltration, and muscle atrophy but it can compromise the tendon healing and increase cuff retears [25,26]. The traditional protocol instead can lend to correct healing but may increase the risk of shoulder stiffness [27] that is the most common complication of rotator cuff repair and a source of pain, functional limitation, and impairment [28]. Therefore, considering these premises and the debate on this topic, the aim of our umbrella review was to investigate the effectiveness of early rehabilitation protocol compared with the traditional one for the following outcome: pain, functional recovery, and risk of retear. Specifically, this paper looks for an answer to the question: “After a rotator cuff repair, is it possible to choose between an early or a traditional protocol according to the patient’s characteristics? If not, could the rehabilitation protocol be based only on the demonstrated efficacy”?

## 2. Materials and Methods

We reported this umbrella review according to the Preferred Reporting Items for Overviews of Reviews (PRIOR) [29].

### 2.1. Eligibility Criteria

The Population, Intervention, Comparison, and Outcome (PICO) method was selected to arrange this review [30].

*Population*: patients with rotator cuff tear undergoing surgical repair, over 18 years old;
*Intervention*: early rehabilitation protocol;
*Comparison*: standard/delayed rehabilitation protocol;
*Primary outcome*: pain;
*Secondary outcome*: function (range of motion, strength) and risk of retear.

#### 2.1.1. Inclusion Criteria

We included systematic reviews, with or without meta-analysis, comparing the efficacy of early rehabilitation protocol with the traditional one after rotator cuff surgical repair, published between 2012 and 2022, in English language and with available full text, reporting outcomes for at least one parameter among pain, shoulder functional and retear rates, with a clinically relevant follow-up time ranging from 3 to 24 months. The definitions of early rehabilitation and traditional rehabilitation were used as described in each study.

#### 2.1.2. Exclusion Criteria

We excluded studies with different aims, published before 2012 and studies that considered tears caused by traumatic events.

#### 2.1.3. Information Sources and Search Strategy

Search strategy was independently applied by three independent reviewers. 

The main MeSH terms and keywords used were: rotator cuff, arthroscopy, shoulder, shoulder joint, rehabilitation, physiotherapy, and physical therapy. The search was conducted in these databases: PubMed, EMBASE, Cochrane Library, PEDro, SCOPUS, and Web of Science (WoS).

#### 2.1.4. Selection Strategy

The data extracted and summarized by three independent reviewers were: name of the authors and year of publication, design of the primary studies included, inclusion criteria of the primary studies, intervention group and comparison with the primary study, tools used to evaluate the results for variables of interest (Pain, ROM, functional scale scores, and retear rate), and primary study references. The data collection process was performed through the reading of full texts and their relevant data were inserted in tables.

#### 2.1.5. Methodological Quality

Three reviewers independently completed assessments of the methodological quality of the included systematic reviews via the AMSTAR-2 [31] and any disagreements were discussed until consensus was reached. AMSTAR-2 is a checklist for the evaluation of systematic reviews, randomized controlled trials and non-randomized studies focusing on health care interventions effectiveness. It consists of 16 Items with the following answer options: “Yes”, “No”, “Yes, in part”. The AMSTAR-2 model is not intended to generate an overall score; however, a score of 1 was assigned to each item if the answer was “Yes”, while the score is null if other answers were given. The quality of the systematic reviews is established on three levels: 0–5 Low, 6–10 Medium, and 11–16 High.

## 3. Results

**Figure 1:**
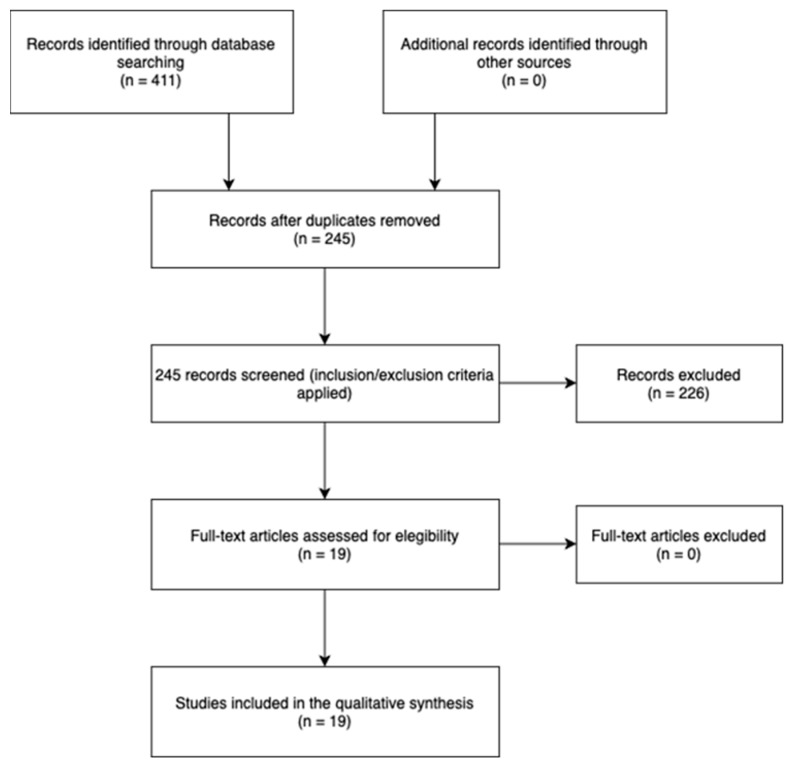
Flowchart.

A total of 19 systematic reviews were included (Figure 1), comparing Early rehabilitation Protocol (EP) versus Traditional rehabilitation Protocols (TP). Where detectable, we reported on the text the information about the size of the lesions and the surgical techniques. Table 2 shows the methodological quality of the included reviews, assessed according to the AMSTAR-2 criteria [31], while Table 3 shows the characteristics of the included reviews. 

The systematic review of Bandara et al. includes six Randomized Controlled Trial (RCT) for a total of 531 patients undergoing either early or delayed rehabilitation protocol after rotator cuff repair. A total of 42 patients with stage 2 or 3 full thickness tear underwent arthroscopic side to side surgery, 124 patients with full thickness tear < 30 mm in width underwent arthroscopic double row surgery, 130 patients with small to large tear size underwent arthroscopic single or double row surgery, 103 patients with full thickness tear underwent arthroscopic single row surgery, the data about the remaining 132 patients were not reported. The results suggest a major functional outcome in the EP, durable for the first six months after surgery, but in the long-term this superiority is not so evident. No statistical significance was found for the recurrence retear risk after EP [32]. Houck et al. collected seven RCTs, that included a population with an average age of 46–59 years, showing a better ROM in the patients submitted to the EP: this lends to a reduction in recovery time but an increased risk of recurrence [33]. The meta-analysis by Li et al. put together eight RCT including 92 patients with partial and full thickness tear, undergoing single or double row arthroscopic surgery, 68 patients with full thickness tear undergoing transosseous equivalent suture-bridge, 105 patients with full thickness tear (<3 cm) undergoing single row or double row or suture-bridge fixation, 64 patients with full thickness tear (1–5 cm) undergoing single row arthroscopy, 114 patients with full thickness tear (<3 cm) undergoing single row surgery, 40 patients with full thickness tear (1–5 cm) undergoing side-to-side repair, and 130 patients with full thickness tear (<5 cm). Different outcomes were analyzed: ROM, evaluated in terms of Forward Flexion (FF) and External Rotation (ER), proved to be totally better in the EP group at mid-term, while at long term only the FF remained superior. For small and medium tear, no differences were discovered in tendon healing, while for large tear TP obtained better results. TP showed superiority in the function outcome also [34]. Littlewood et al. analyzed 12 RCT, including 819 patients with any size of tear, repaired arthroscopically, with an average age of 58.1, reporting no significant differences between the two protocols in terms of function and retear rates [35]. Longo et al. underlined that EP obtained better results in external rotation at 3 and 6 months, while at 24 months the result was the same as TP. No differences were found in the retear rate [36]. The review by Longo et al. focused on retear rates after rotator cuff surgery, showing no statistical difference among the different period of immobilization [37]. In the systematic review by Matlak et al., only 13 studies focused on the protocols’ different outcomes, showing similar long-term results achieved with both early and delayed mobilization. Those studies included 264 patients with unspecified tear size and technique, 206 patients with full thickness tear, undergoing single, double row, suture bridge or transosseous repair, 30 patients with 1–3 cm tear, undergoing single row repair, 73 patients with full thickness tear, undergoing transosseous-equivalent repair with PEEK, 64 patients with medium or large tear, undergoing arthroscopic single row repair, 100 patients with 2–4 cm tear, undergoing arthroscopic single row repair, 40 patients with unspecified tear size, undergoing sing row arthroscopic repair, 68 patients with full thickness tear, undergoing transosseous-equivalent suture-bridge technique and 199 patients with tear of any size, undergoing single or double row repair. Following the literature, EP may decrease the risk of stiffness and quickly improve the ROM, while TP should reduce the risk of retear. Furthermore, the early isometric loading in the EP can reduce pain: the authors sustained that the stimulation of scar and tendon may contribute to improve this outcome [38]. Mazuquin et al. found no differences between the two protocols concerning pain, function, and tendon healing; otherwise, they noticed a better short-term and long-term ROM, especially regarding: shoulder flexion at six weeks, three–six months and one year follow-up, abduction at six weeks follow-up, external rotation at three–six months follow-up, internal rotation at six weeks, three–six months follow-up [12]. In the work by Saltzman et al., eight studies showed a high level of evidence that EP can let the patient achieve an extended ROM up to 1 year, but it may result in greater retear rates [39]. Silveira et al. included 132 patients with mean tear size, undergoing unspecified surgical technique, 14 patients with unspecified tear size and technique, 98 patients with unspecified tear size, undergoing arthroscopic repair, 189 patients with any size of tear, undergoing mini-open repair, 206 patients with mean size tear, undergoing single or double row repair, 118 patients with mean size tear, undergoing single row arthroscopic repair, 29 patients with medium and large size, undergoing side to side repair. They found that patients who started active shoulder movement early after rotator cuff repair had greater shoulder range of motion in an initial stage, but the long-term results are comparable. However, the group differences did not appear to be clinically important, and rotator cuff integrity was similar [40]. The findings of the review by Thomson et al., which included 706 patients with an average age of 58.1 years, suggest that there may not exist a better rehabilitation protocol, so the EP and the TP are comparable [41]. Gallagher et al. analyzed 8 RCTs, including 105 patients with small to medium full thickness tear, undergoing single or double repair; 93 patients with medium to large full thickness, undergoing single row repair; 100 patients with partial or full thickness, undergoing single or double row repair; 68 patients with full thickness tear, undergoing suture bridge repair; and 114 patients with small to medium full thickness tear, undergoing double row repair, finding that the EP may provide an initial improvement in ROM and function, but the outcome at one year is similar to the one obtained with the TP. Furthermore, the EP may sustain a major risk of retear in larger tears [42]. Chang et al. stated that the EP can reduce the postoperative stiffness after arthroscopic repair but in larger tears may not guarantee a correct healing [43]. The systematic review by Chan et al. did not identify any difference in outcome after arthroscopic repair for function, ROM, and recurrency of tear [44]. Shen et al. included 68 patients with full thickness tear, undergoing transosseous equivalent suture bridge; 92 patients with partial or full thickness undergoing single or double row repair; 105 patients with small to medium full thickness tear, undergoing single or double row or suture bridge, and they could not prove that EP could represent a higher risk of tendon healing. Secondly, they found out that shoulder ROM in the EP was faster regained [45].

Huang et al. analyzed 100 patients with partial thickness tear, undergoing arthroscopic repair, 29 patients with partial thickness tear, undergoing side to side repair, 68 patients with full thickness tear undergoing arthroscopic suture bridge, 95 patients with small to medium tears undergoing single or double row or suture bridge repair, 64 patients with medium to large tear undergoing arthroscopic single row repair, and 92 patients with partial or full thickness tear undergoing single or double row repair. They found in the EP group a better achievement in ROM and shoulder function, but the early rehabilitation may increase the risk of retear and bad tendon healing. In the EP, pain outcome was better in the first weeks of treatment, but no differences were found at six or twelve months follow up [46]. The five studies included in the Riboh et al. review show that after arthroscopic repair of different tear size the EP achieves a better short-term and long-term result for ROM after small and medium tears repair while no difference in retear rate is proved among the two protocols [47]. Kluczynski et al. focused on the effect of passive ROM exercises after rotator cuff repair, finding some interesting differences regarding tendon healing linked to the tear size; with the early protocol, risk of retear is lower for tears smaller than three centimeters but it appears to be higher for tears larger than five centimeters [48]. Finally, Kluczynski et al. evaluated the effect of starting active ROM exercises in two different times of rehabilitation protocol, reporting that EP had negative effects on tendon healing when applied in patients with rotator cuff tears smaller than three centimeters and larger than five centimeters [49].

## 4. Discussion

Since the aim of this umbrella review was to examine the effectiveness of post-surgical rotator cuff repair rehabilitation protocol (early or traditional), we decided to divide the discussion into three main points: pain, functional recovery, and risk of retear.

### 4.1. Pain

Most of the included systematic reviews showed that there was no significant difference in pain relief between the early rehabilitation and traditional rehabilitation protocols [35,40,42]. However, one study reported that early mobilization might lead to moderate better pain relief in the short term [46], while long-term pain relief (about 3–4 months after surgery) was comparable between the two protocols. Since pain can often arise from postoperative shoulder stiffness, early protocol may be a helpful rehabilitation technique to prevent the stiffness deriving from shoulder immobilization. On the other hand, an early isometric loading and stimulation of tendon and scars (as realized with EP) may represent another mechanism for pain reduction. 

### 4.2. Functional Recovery

Functional recovery, concerning range of motion, strength, and quality of life, was one of the key aspects examined in this review. The findings showed that early rehabilitation protocols provide quicker improvements in range of motion [32,33,34,39,40,42,43] particularly in the first 6 months after surgery. This faster recovery represents an advantage as it may lead to a rapid return to normal daily life and can also impact quality of life as the patient can return to working activities and social activities. However, some studies report that these advantages in ROM might not persist in the long term [42,43]: basically, it means that EP may provide a faster initial recovery, but the ultimate outcome would be similar between the two protocols. Regarding other functional scores, such as the Constant-Murley Shoulder Score and the American Shoulder and Elbow Surgeons (ASES) score, there is no consistent evidence to suggest that early or delayed rehabilitation protocols provide significantly better outcomes [12,35,37,39,40,41,42,44]. In terms of strength, most of the included studies suggested no significant differences between EP and TP [12,39,40]. Only the review by Matlak et al. showed improvements in external rotation strength using an EP [38]. In conclusion, it remains essential to customize the timing and progression of strengthening exercises on the patient’s needs, also considering the subsequent therapy response.

### 4.3. Risk of Retear

Risk of retear represents a crucial disadvantage in early rehabilitation protocols, as stated in some of the analyzed systematic reviews [32,33,34,37,38,39,43]. On the contrary, other studies reported no significant differences in recurrence rates between the two rehabilitation approaches [12,36,39,44,47]. The main factor influencing the risk of retear seems to be the size of the tear: patients with 3 to 5 cm and 5 or more-centimeters tear sizes undergoing EP rehabilitation are those with a higher risk of recurrency among the total [33,42,43]. Only Kluczynski et al. found a higher risk for patients in the EP group with rupture < 3 cm when repaired with transosseous and single-row suture anchor techniques [49]. A possible explanation for the increased risk of retear in EP can be found in the early mobilization and loading of the repaired tendon, which might compromise the healing process. On the other hand, the traditional protocol allows the tendon to heal in a longer time before starting active movements, reducing the risk of retear. In some cases, based on our study, a conservative approach seems be suitable for patients with large tears and a higher risk of retear, while early rehabilitation could be better for patients with smaller tears who are seeking a quicker return to their daily activities. An important factor to consider is the recovery time: the patient may incur hospitalization-related diseases if the recovery period is extended and feel disadvantaged if he does not return to normal activities immediately. This suggests that the EP can be useful in reducing the recovery time and the derived expenses.

More comparisons based on other patient characteristics, such as age, smoking habit, gender, and occupation would be useful to define increasingly personalized and effective rehabilitation protocols. Further research is needed to establish the most effective rehabilitation strategies for different patients and injury characteristics.

This study is primarily limited by the fact that we did not report all the surgical techniques used for the repair, often missing in the articles included in the review. In particular, large, retracted tears of the cuff often exclude the double row technique which has proven to have superior biomechanical properties especially in the initial phase of healing. For this reason, in fact, this represents the major limitation of our article. Secondly, the size of the lesions was also not always reported by all the articles we included, as was the adherence to different rehabilitation protocols. This information, not reported by the authors, represents a gap that requires future studies. Thirdly, we decided to focus this work only on chronic tears because collecting the data of acute and chronic conditions together would have created too much dispersion since they have different outcomes [50,51].

## 5. Conclusions

This umbrella review showed that both early and delayed rehabilitation protocols after arthroscopic rotator cuff repair surgery can provide adequate pain relief and functional recovery. Early rehabilitation protocols generally lead to better short-term ROM outcomes and strength improvement, potentially. However, these advantages may not persist in the long term. The fastest recovery provided by the EP may bring a reduction in the costs of medical assistance for both patient and medical system. Risk of recurrence remains a concern for early rehabilitation, particularly for large injuries; clinicians should carefully consider the patient’s individual characteristics, injury severity and specific therapy modalities when determining the most appropriate rehabilitation protocol after rotator cuff repair. Based on the information collected, the only patient-related characteristic that can be useful to guide the choice between the two protocols is the size of the lesion. Future studies are needed to better quantify the possible differences and characteristics that can influence the choice of one protocol over another.

## Figures and Tables

**Table 1:** Example of early vs. delayed protocol.

Week	Early Protocol	Delayed Protocol
0–3	- Passive forward elevation 0°−120°, 3× weekly with a physical therapies - Passive external rotation 0°−30°, 3× weekly with a physical therapist - Pendulum exercises, 3× daily for 5 min per session - Active elbow, wrist, and hand ROM	- Pendulum exercises, 3× daily for 5 min per session - Active elbow, wrist, and hand ROM
4–6	- Passive forward elevation to tolerance, 3× weekly with physical therapist - Passive external rotation 0°−45°, 3× weekly with physical therapist - Pendulum exercises, 3× daily for 5 min per session - Active elbow, wrist, and hand ROM	- Pendulum exercises, 3× daily for 5 min per session - Active elbow, wrist, and hand ROM
6–10	- Active assisted ROM, 3× weekly with a physical therapist	- Passive forward elevation to 120°, 3× weekly - Passive external rotation to 30°, 3× weekly - At week 7, progress to passive forward elevation to tolerance and 45° external rotation - At week 7, begin active assisted ROM 3× weekly with physical therapist
10–12	- Active assisted ROM 3× weekly with a physical therapist - Active ROM to tolerance	- Active assisted ROM 3× weekly with a physical therapist - Active ROM to tolerance

**Table 2:** AMSTAR-2 criteria.

Authors	q1	q2	q3	q4	q5	q6	q7	q8	q9	q10	q11	q12	q13	q14	q15	q16
**Bandara et al.** [32] (**2021**)	yes	partial yes	yes	partial yes	yes	yes	no	partial yes	no	no	yes	no	no	yes	no	no
**Houck et al.** [33] (**2017**)	no	partial yes	yes	partial yes	yes	yes	no	partial yes	no	no	yes	no	yes	yes	no	yes
**Li et al.** [34] (**2017**)	yes	partial yes	yes	partial yes	yes	yes	Partial Yes	yes	yes	no	yes	yes	yes	yes	yes	yes
**Littlewood et al.** [35] (**2014**)	yes	partial yes	yes	partial yes	no	no	no	yes	no	yes	no-meta	no-meta	no	no	no-meta	no
**Longo et al.** [36] (**2021**)	yes	partial yes	yes	partial yes	yes	yes	no	yes	yes	yes	yes	yes	yes	yes	yes	yes
**Longo et al.** [37] (**2021**)	yes	partial yes	yes	partial yes	yes	yes	no	yes	yes	no	yes	yes	yes	yes	yes	yes
**Matlak et al.** [38] (**2021**)	no	partial yes	yes	partial yes	yes	yes	no	yes	no	no	no-meta	no-meta	yes	no	no-meta	yes
**Mazuquin et al.** [12] (**2021**)	yes	partial yes	yes	partial yes	yes	yes	yes	yes	yes	no	yes	yes	yes	yes	yes	yes
**Saltzman et al.** [39] (**2017**)	no	partial yes	yes	partial yes	yes	yes	no	no	no	yes	no-meta	no-meta	no	yes	no-meta	no
**Silveira et al.** [40] (**2021**)	yes	partial yes	yes	partial yes	yes	yes	partial yes	yes	yes	yes	yes	yes	yes	yes	yes	no
**Thomson et al.** [41] (**2015**)	yes	partial yes	yes	yes	yes	yes	partial yes	partial yes	no	no	no-meta	no-meta	no	no	no-meta	yes
**Gallagher et al.** [42] (**2015**)	yes	yes	yes	yes	no	no	no	yes	yes	no	no meta	no meta	yes	yes	no meta	yes
**Chang et al.** [43] (**2014**)	yes	yes	yes	yes	yes	yes	partial yes	yes	yes	no	yes	yes	yes	yes	yes	yes
**Chan et al.** [44] (**2014**)	yes	yes	yes	yes	yes	yes	yes	yes	partial yes	no	yes	yes	no	yes	yes	no
**Shen et al.** [45] (**2014**)	yes	yes	yes	yes	yes	yes	no	partial yes	yes	no	yes	yes	no	yes	yes	no
**Huang et al.** [46] (**2013**)	yes	partial yes	yes	yes	yes	no	no	yes	no	no	no	yes	no	yes	no	no
**Riboh et al.** [47] (**2014**)	yes	yes	yes	yes	yes	yes	no	yes	yes	no	no	yes	yes	yes	yes	yes
**Kluczynski et al.** [48] (**2014**)	yes	yes	yes	yes	no	no	no	partial yes	no	no	yes	no	no	yes	no	yes
**Kluczynski et al.** [49] (**2015**)	yes	yes	yes	yes	yes	yes	no	yes	no	no	no	no	no	yes	no	yes

**Table 3:** Characteristics of the included reviews.

	Article Type	Population	Evaluation Time	Results	Outcomes and Scales	Conclusions	Limitations
**Bandara et al.** [32]	Systematic review (6 RCT)	531 patients Diagnosis: All participants received rotator cuff repair Average age and DS: non specified. M = non specified F = non specified	T0 = first postoperative day T1 = 6 month after rotator cuff repair T2 = 12 month after rotator cuff repair	=ROM EP > Constant-Murley Score (no increased risk of recurrence)	Joints balance = ROM. Function = Constant-Murley Shoulder Score. Structure = Recurrence rate	(1) EP = TP—ROM. (2) EP > risk of recurrence but > functional recovery (3) EP = TP—safe and reproducible results in the short and long term.	1 Variable design of each individual study. 2 High heterogeneity revealed in pooled analyses. 3 Variability in the description of each rehabilitation protocol and timing 4 Possibility of bias in a number of the included studies
**Houck et al.** [33]	Systematic review (7 RCT)	5896 patients Diagnosis: All participants received rotator cuff repair Average age: 46–59 years DS: non specified M = non specified F = non specified	T0 = first postoperative day T1 = 6 month after rotator cuff repair T2 = 12 month after rotator cuff repair T3 = 24 month after rotator cuff repair	EP > ROM EP > risk of recurrence TP > Cure rate TP > ASES score EP > small injuries TP > large injuries	joints balance = ROM. Function = ASES score. Structure = Recurrence rate	(1) EP > ROM but >risk of recurrence	1 lack of reporting follow-up results, age, sex, tear size, and the rotator cuff muscles involved. 2 surgical techniques inconsistently reported in the included studies. 3 risks of bias in the ROM reported due to a lack of blinding.
**Li et al.** [34] (**2017**)	Systematic review (8 RCT)	671 patients Diagnosis: All participants received rotator cuff repair Average age: 58.1 ± 3.9 DS M = non specified F = non specified	T0 = first postoperative day T1 = 3 month after rotator cuff repair T2 = 6 month after rotator cuff repair T3 = 12–24 month after rotator cuff repair	EP > ROM =cure rate, ASES at T2, SST, Constant-Murley score TP > ASES at T3	joints balance = ROM. Function = Constant-Murley Shoulder Score, ASES, SST. Structure = Recurrence rate	(1) EP > ROM but < shoulder functionality (2) EP < cure rate for large injuries	1 number of trials relatively small 2 no high quality of evidence in all outcomes 3 outcome assessors were not blinded to rehabilitation protocol. 4 the standard deviation is not provided in some included studies.
**Littlewood et al.** [35] (**2014**)	Systematic review (12 RCT)	819 patients Diagnosis: All participants received rotator cuff repair Average age: 58.1 DS: non specified M = 430 F = 389	T0 = first postoperative day T1 = 3 month after rotator cuff repair T2 = 6 month after rotator cuff repair T3 = 12 month after rotator cuff repair	=pain, risk of recurrence and disability	Function = pain, disability Structure = Recurrence rate	(1) EP = TP	1 small mean number of included participants per trial 2 only one reviewer identified relevant studies, extracted data, and synthesized the findings.
**Longo et al.** [36] (**2021**)	Systematic review (16 RCT)	1424 patients Diagnosis: All participants received rotator cuff repair Average age: 56.1 ± 8.7 DS PP56.6 ± 9 DS PT M = 776 F = 648	T0 = first postoperative day T1 = 3 month after rotator cuff repair T2 = 6 month after rotator cuff repair T3 = 12 month after rotator cuff repair T4 = 24 month after rotator cuff repair	EP > ROM external rotation at T1. EP > ROM T2. =ROM at T4. =risk of recurrence and Constant-Murley score	joints balance = ROM. Function = Constant-Murley Shoulder Score. Structure = Recurrence rate	(1) = recurrence rate between the 2 groups (2) EP > external rotation at 3- and 6-months follow-up, but = at 24	1 lack of information on the RC tear characteristics 2 muscle atrophy and fatty infiltration were not specified in most of the included articles. 3 Different early protocols in terms of exercise and timing
**Longo et al.** [37] (**2021**)	Systematic review (31 RCT)	5109 patients Diagnosis: All participants received rotator cuff repair Average age: 58.2 years ± 3.7 DS M = 2396. F = 2231	T0 = first postoperative day T1 = 3 month after rotator cuff repair T2 = 6 month after rotator cuff repair T3 = 12 month after rotator cuff repairT4 = 24 month after rotator cuff repair	=immobilization period =passive ROM EP active ROM > risk of recurrence TP complete active ROM > risk of recurrence =strengthening exercises	Structure = Recurrence rate	(1) = recurrence rate for immobilization, passive ROM, and force exercises. (2) EP active ROM > recurrence rate (3) TP full active ROM > recurrence rate	1 insufficient number of studies reporting the preoperative tear size. 2 no conclusions regarding clinical outcomes were made.
**Matlak et al.** [38] (**2021**)	Systematic review (22 RCT)	1782 patients	T0 = first postoperative day	EP > ROM;	joints balance = ROM;	(1) EP = reduced risk of stiffness, improves ROM and function faster	1 Lack of high quality studies about subscapularis rehabilitation
		Diagnosis: All participants received rotator cuff repair	T1 = 6 weeks after rotator cuff repair	EP > Function	Structure = Recurrence rate, rigidity	(2) TP = Reduced risk of recurrence.	
		Average age: 45–64.8 years	T2 = 3 month after rotator cuff repair	EP < Rigidity	Structure = strength	(3) CPM can accelerate ROM gain but does not improve long-term results.	2 Literature gaps about optimal dosage of frequency and intensity of exercise, ideal time to begin loading.
		DS: non specified	T3 = 6 month after rotator cuff repair	TP < risk of recurrence		(4) Early isometric loading may be beneficial for increasing strength and tendon shaping but requires further research	
		M = non specified		EP > strength			
		F = non specified					
**Mazuquin et al.** [12] (**2021**)	Systematic review (20 RCT)	1841 patients	T0 = first postoperative day	=VAS;	joints balance = ROM;	(1) EP > ROM and same tendon integrity	1 The majority of the RCTs were considered of high or unclear overall risk of bias, had small sample sizes and their definition of early and delayed rehabilitation were not consistent
		Diagnosis: All participants received rotator cuff repair	T1 = 6 weeks after rotator cuff repair	=ASES, Constant-Murley, SST, WORC;	Function = ASES, Constant-Murley Shoulder Score, SST, WORC; SANE;		2 subgroup analyses were not possible due to the lack of data reported by tear size
		Average age: 54–65.4 years	T2 = 3 month after rotator cuff repair	EP > SANE Score;	Structure = strength, tendon integrity		
		DS: non specified	T3 = 6 month after rotator cuff repair	=Strength, tendon integrity			
		M = non specified	T4 = 1 year after rotator cuff repair	EP > ROM short term			
		F = non specified	T5 = 2 years after rotator cuff repair	TP > rigidity long term			
**Saltzman et al.** [39] (**2017**)	Systematic review (9 RCT)	265 −2251 patients	T0 = first postoperative day	=tendon healing, risk of recurrence, functional outcomes, and strength	joints balance = ROM;	(1) EP > ROM	Difficulty in controlling for heterogeneity, small sample sizes and narrow study populations, lack of blinding in individual studies
		Diagnosis: All participants received rotator cuff repair	T1 = 6 month after rotator cuff repair	EP > ROM;	Function = ASES, Constant-Murley, SST, WORC;	(2) = Functional results and recurrence rate	
		Average age: 57.7–60.38 years	T2 = 12 month after rotator cuff repair	EP > risk of recurrence for large injuries	Structure = Recurrence rate and recovery rate	(3) EP > Recurrence rate for large injuries	
		DS: non specified					
		M = non specified					
		F = non specified					
**Silveira et al.** [40] (**2021**)	Systematic review (8 RCT)	756 patients	T0 = first postoperative day	=pain, strength, and integrity	joints balance = ROM;	(1) EP > freedom of movement of the shoulder but worse quality of life;	Different tear size and surgical techniques
		Diagnosis: All participants received rotator cuff repair	T1 = 6 weeks after rotator cuff repair	TP > WORC Index at T1,	Function = WORC Index, Constant-Murley score;	(2) Differences between groups do not appear to be clinically important	
		Average age: 50.43–57.68 years	T2 = 3 month after rotator cuff repair	=in other follow-up times	Structure = strength, tendon integrity		
		DS: non specified	T3 = 6 month after rotator cuff repair	=Constant-Murley score;			
		M = 442;	T4 = 1 year after rotator cuff repair	EP > ROM at T1, = in other follow-up times			
		F = 344.	T5 = 2 years after rotator cuff repair				
**Thomson et al.** [41] (**2015**)	Systematic review (11 RCT)	706 patients	T0 = first postoperative day	EP > ROM	joints balance = ROM	(1) EP = TP	1 Data extracted by only one reviewer 2 Language and publication bias
		Diagnosis: All participants received rotator cuff repair	T1 = 6 month after rotator cuff repair	TP > large injuries			
		Average age: 58.1 years	T2 = 12 month after rotator cuff repair				
		DS: non specified					
		M = non specified					
		F = non specified					
**Gallagher et al.** [42] (**2015**)	Systematic review (6 RCT)	80 patients	T0 = first postoperative day	=risk of recurrence, PAIN	Function: Constant shoulder score, ASES, SST, UCLA, and DASH score	EP better ROM in short term, but =in long term	1 lack of uniform, prospective trials comparing similar rehabilitation protocols 2 All studies suffered from an intrinsic inability to properly blind individuals and several suffered from inadequate randomization or insufficient incomplete outcome reporting
		Diagnosis: All participants received rotator cuff repair	T1 = 3 month after rotator cuff repair	EP > ROM at T2, = ROM at T3			
		Average age: 54.5–63.2	T2 = 6 month after rotator cuff repair	=stiffness, =healing			
		DS: non specified	T3 = 12 month after rotator cuff repair	=ASES, SST, DASH			
		M = non specified		EP > UCLA at T1, but = at T2 and T3			
		F = non specified					
**Chang et al.** [43] (**2014**)	Systematic review (6 RCT)	482 patients	T0 = closest day to surgery	=external rotation range	Function = UCLA and Constant	Early ROM exercises improve postoperative stiffness but improper tendon healing in large-sized tears	1 Small numbers of included trials
		Diagnosis: All participants received rotator cuff repair	T1 = 6 month after rotator cuff repair	EP > shoulder forward flexion range at T1 and T2			2 heterogeneities among the included articles regarding the severity of the rotator cuff tears, surgical techniques, and functional outcome assessment scales
		Average age: 54.5–63.5	T2 = 12 month after rotator cuff repair	EP > recurrency	Structure = recurrence rate		3 not all the included trials reported
		DS: non specified		EP = reduce stiffness			reoperation rate
		M = 233					
		F = 249					
**Chan et al.** [44] (**2014**)	Systematic review (4 RCT)	370 patients	T0 = closest day to surgery	=ASES (4 RCT), CMS (2 RCT), SST, WORC	Function = ASES, Constant, SST, WORC, DASH	No statistically significant differences in functional outcomes scores, relative risks of recurrent rotator cuff tears	1 Unavailable data for several studies included in the review.
		Diagnosis: All participants received rotator cuff repair	T1 = latest time point in all trials	=recurrence	Structure = recurrence rate		2 None of the outcomes were judged to be of high quality by the author
		Average age: 65		=ROM	Joint balance = ROM		3 Lack of blinding
		DS: non specified					
		M = 203					
		F = 167					
**Shen et al.** [45] (**2014**)	Systematic review (3 RCT)	265 patients	T0 = day one postoperatory	EP > Constant (1 RTC) at 12 months	Function = ASES, Constant, SST	No significant differences in tendon healing. EP > external rotation at six moths but no at 1 year	1 small number of rcTs included
		Diagnosis: All participants received rotator cuff repair	T1 = 6 months	=ASES, SST e VAS	Pain = VAS	EP Fastest ROM recovery	2 some clinical heterogeneity among trials
		Average age: 55.3–63.5	T2 = 12 months	=tendon healing			
		DS: non specified		=ROM			
**Huang et al.** [46] (**2013**)	Systematic review (6 RCT)	448 patients	T0 = day one postoperatory	EP > ROM	Function: DASH, Constant, ASES, SST	EP > ROM but greater risk of un-healing or re-tearing	1 few article with variable outcome measures and time points of follow-up
		Diagnosis: All participants received rotator cuff repair	T1 = 6 months	EP > function	Pain = VAS		2 data of some studies did not fit normal distributions and could not be calculated
		Average age: 55–63	T2 = 12 months	TP > healing	Structure = healing		
		DS: non specified		EP > risk of retear			3 all articles were only of fair quality
				EP > VAS at week 5 and 16, but EP = TP			
				at T1 and T2			
**Riboh et al.** [47] (**2014**)	Systematic review (5 RCT)	451 patients	T0 = day one postoperatory		Function = Constant, SST, ASES, UCLA	EP > shoulder forward flexion at 3/6/12 months, external rotation only at 3 months	1 methodologic limitations and moderate risk of bias of 3 of the 5 randomized studies included
		Diagnosis: All participants received rotator cuff repair	T1 = 3 months	=recurrence	Pain = VAS	=recurrency	2 all of the studies suffered from performance bias because neither surgeons nor patients could be blinded to the treatment-group assignment.
		Average age: 54.8–63.2	T2 = 6 months	EP > ROM	Structure = healing		3 All 5 studies provide only Level II data
		DS: non specified	T3 = 12 months				
**Kluczynski et al.** [48] **2014**	Meta Analysis (28 RCT)	1729 patients	T0 = day one postoperatory	EP > risk of retear >5 cm	Structure = recurrence rate, healing	EP greater risk of retear for >5 cm tears, TP greater risk of <3 cm tears	1 RC healing as only outcome examined
		Diagnosis: All participants received rotator cuff repair	T1 = latest time point in all trials	TP > risk of retear <3 cm			2 Most studies included in this review provided evidence levels of 2 to 4
							3 Focused only on passive ROM
		Average age: non specified					
		DS: non specified					
**Kluczynski et al.** [49] **2015**	Meta Analysis (37 RCT)	2251 patients	T0 = day one postoperatory	EP > risk of retear	Structure = recurrence rate, healing	EP greater risk of retear for >5 cm tears ad <3 cm tears	1 RC healing as only outcome examined
		Diagnosis: All participants received rotator cuff repair	T1 = latest time point in all trials, at least 1 year				2 focused only on the active ROM component of rehabilitation
							3 unable to control for the heterogeneity of these studies
		Average age: non specified					4 small sample size of the early active ROM group
		**DS: non specified**					

RCT: Randomized Controlled Trial; ROM: Range Of Motion; EP: Early Protocol; TP: Traditional Protocol; ASES: American Shoulder and Elbow Society; CPM: Continuous Passive Motion; WORC Index: Western Ontario Rotator Cuff Index; SST: Simple Shoulder Test; SANE: Single Assessment Numeric Evaluation; VAS: Visual Analalogic Scale; UCLA: University of California Los Angeles; DASH: Disabilities of the Arm, Shoulder and Hand; CMS: Constant-Marley Scale.

## Data Availability

In this study no data were reported.
